# Are Treated Celiac Patients at Risk for Mycotoxins? An Italian Case-Study

**DOI:** 10.3390/toxins9010011

**Published:** 2016-12-28

**Authors:** Martina Cirlini, Teresa Mazzeo, Leda Roncoroni, Vincenza Lombardo, Luca Elli, Maria T. Bardella, Carlo Agostoni, Luisa Doneda, Furio Brighenti, Chiara Dall’Asta, Nicoletta Pellegrini

**Affiliations:** 1 Department of Food Science, University of Parma, Parco Area delle Scienze, 49/A, Parma 43124, Italy; martina.cirlini@unipr.it (M.C.); teresa.mazzeo@studenti.unipr.it (T.M.); furio.brighenti@unipr.it (F.B.); nicoletta.pellegrini@unipr.it (N.P.); 2 Center for Prevention and Diagnosis of Celiac Disease, Gastroenterology and Endoscopy Unit, Department of Pathophysiology and Transplantation, University of Milan, Fondazione IRCCS Cà Granda Ospedale Maggiore Policlinico, Milan 20122, Italy; l.roncoroni@nutrizionista.la (L.R.); vicky.l@hotmail.it (V.L.); lucelli@yahoo.com (L.E.); mariateresa.bardella@yahoo.com (M.T.B.); 3 Department of Biomedical, Surgical and Dental Sciences, University of Milan, Milan 20122, Italy; luisa.doneda@unimi.it; 4 Intermediate Pediatric Care Unit, IRCCS Ca’ Granda, Ospedale Maggiore Policlinico, Milan 20122, Italy; agostoc2@gmail.com; 5 Department of Clinical Sciences and Community Health, University of Milan, Milan 20122, Italy

**Keywords:** deoxynivalenol (DON), zearalenone (ZEN), fumonisin B1 (FB1), human urine, celiac patients

## Abstract

Urinary biomarkers of mycotoxin exposure were evaluated in a group of celiac patients (*n* = 55) and in a control group of healthy subjects (*n* = 50) following their habitual diet. Deoxynivalenol (DON), zearalenone (ZEN), and fumonisin B1 (FB1) were monitored in 105 urinary samples collected from the two groups. Dietary habits were also recorded through compilation of a seven-day weighed dietary diary. Biomarkers of mycotoxin exposure were detected in 21 celiac patients and in 15 control subjects, corresponding to about 34% of total participants. In particular, ZEN was the most detected mycotoxin among all the studied subjects with a total of 19 positive cases. Results did not show a statistically significant difference in mycotoxin exposure between the two groups, and the presence of specific mycotoxins was not related to the intake of any particular food category. Our findings suggest little urgency of specific regulation for gluten free products, although the prevalence of exposure observed in free-living diets of both celiac and healthy subjects underlines the need of a constant surveillance on mycotoxins occurrence at large.

## 1. Introduction

Cereals and cereal-based foods are staple foods all over the world, especially in the Mediterranean countries. The most recent Italian food consumption survey, conducted by INRAN-SCAI in 2005–2006 and involving 3323 subjects, showed that the mean cereal consumption in the general Italian population was around 258 ± 106 g/day per capita and the main sources were bread (103 ± 77 g/day), followed by pasta and pasta substitutes (54 ± 33 g/day), wheat and other cereal flours (36 ± 36 g/day), cakes and sweet snacks (17 ± 28 g/day), and rice (15 ± 24 g/day) [1].

Cereals, especially wheat and maize, are proved to be the main contributors to mycotoxin intake worldwide [2]. Mycotoxins are secondary metabolites produced by filamentous fungi in crops, according to climatic conditions. Due to the high stability towards technological treatments, fungal contamination may be transferred along the production chain, from the field to the raw commodities and then to final products. Among mycotoxins, those produced by *Fusarium* spp. such as deoxynivalenol (DON), zearalenone (ZEN), and fumonisins (FBs) are mainly related to cereals contamination in temperate areas. These compounds are responsible of a wide range of toxic activities—i.e., inflammation and immunosuppression, disruption of sphingolipid metabolism, and disruption of the endocrine system [3]. 

Recently, several studies estimated the mycotoxin exposure through diet [4,5,6,7]. The occurrence of multiple natural toxins in composite foods and drinks was evaluated in the Netherlands in 2013 [8]. Although, at low levels, results showed a wide incidence of regulated and emerging mycotoxins in different commodities, mainly cereals. 

A similar survey on the Belgian market confirmed the widespread occurrence of *Fusarium* mycotoxins in cereal-based products, mainly those based on whole-meal or enriched in fiber [9].

While DON and ZEN can contaminate different grains, fumonisins are mainly associated with maize and products thereof [10]. Due to the warmer climatic conditions, maize crops from Southern Europe are strongly affected by fumonisins accumulation [11]. In consideration of the lower intake of maize compared to other cereals, exposure to fumonisins is often considered low compared to other mycotoxins. However, maize-derived ingredients are frequently used in food formulation. In addition, some population sub-groups may be more exposed compared to the general population. As an example, D’Arco et al. (2009) considered the FBs occurrence in products addressed to children or vegans and celiac persons on the Italian and Spanish markets, reporting a higher incidence of positive samples within the class of organic foods [12]. Among population categories particularly vulnerable to mycotoxins, patients suffering from celiac disease might be potentially overexposed due to their restricted gluten-free (GF) diet. However, very few studies in the literature specifically addressed the mycotoxins exposure of this population group [13,14,15,16,17,18]. The presence of DON was monitored in corn-based foods, in particular in fried and baked corn snacks and in corn-based breakfast cereals collected from supermarkets in Valencia (Spain); more than the 25% of the total samples considered tested positive for DON in a range of 26–132 µg/L [17]. The occurrence of FBs was investigated in different GF products purchased on the markets in Italy in 2009: the findings demonstrated a diffuse occurrence of fumonisins in these special foods. In particular, 82% of the considered samples were contaminated and some samples, in particular corn flours and an extruded products, exceeded the normative limit of 800 µg/L [18]. The presence of fumonisins was also observed during a successive study on different certified corn-based GF products; 88% of the samples were contaminated and 8/118 products showed FB values exceeding the legal limit fixed by the European Commission [14]. Nevertheless, the fumonisin exposure evaluated measuring the Sphinganine/Sphingosine ratio in the urine of 80 subjects, 40 controls, and 40 celiac patients, was similar in the two groups [14]. More recently, an analogous investigation on the presence of different mycotoxins in GF products collected from Italian market showed the presence of FBs and ZEN in all the categories of products taken into account with the exception of pasta. In particular, FBs were found in 29% of the analyzed samples, ZEN in 11%, while low levels of DON were detected in few samples [13].

Although risk assessment is commonly based on the combination of occurrence and consumption data, urinary biomarkers can be of great support when specific vulnerable subpopulation groups are taken into account. The urinary excretion and quantification of mycotoxins, as of their metabolites, could be used to estimate the actual human exposure toward these toxic compounds. So far, several studies monitored urinary biomarkers in population groups from rural areas in developing countries [19,20,21] and, more recently, the same approach was also used to monitor the exposure of the population throughout Europe [4,22,23]. The combined use of biomarkers and dietary intake data has been proposed by Hendricks et al. for a better assessment of mycotoxin exposure in Belgium [4]. Results of the study clearly show that a significant exposure to DON, ochratoxin A, and citrinin exists for a broad segment of the Belgian population. A similar approach was reported by Wallin et al. for the Swedish population [22]. The authors reported that, according to urinary biomarkers, significant exposure to more than one toxin was found in 69% of the study population. However, when comparing the number of toxins detected in urine with the reported consumption data, it was difficult to distinguish food patterns which would indicate an increased risk of exposure to many mycotoxins simultaneously. Concerning Italy, a study on 52 volunteers from Apulia was recently performed by monitoring urinary biomarkers for multiple mycotoxin exposure [24]. While a significant percentage of the subjects exceeded the total daily intake (TDI) for OTA (94%), and DON (40%), the estimated human exposure to fumonisins and ZEN was largely below the TDI for all volunteers.

However, despite the particular relevance that corn-based diets have for celiac patients, no data exist on the urinary biomarkers of exposure in this specific population.

The present study is aimed at evaluating the mycotoxin exposure through urinary biomarkers in a group of celiac patients located in Northern Italy compared to a control group. Among mycotoxins, the fumonisins DON and ZEN were considered, on account of the formulation of GF products. In addition, data collected on urinary biomarkers were compared to dietary intake records in order to identify the contribution of the most likely food sources of mycotoxin.

## 2. Results

### 2.1. Dietary Data

Celiac patients and control subjects enrolled had similar baseline characteristics and daily energy intakes, as shown in Table 1.

As expected, celiac patients selected their foods in large majority in the form of GF products. Table 2 reports the mean and median daily consumption of food items for celiac patients and control subjects. Regarding the food groups, celiac patients ate significantly less bread and substitutes in refined and whole-meal form and flours, but more rice and other cereals than control subjects. Conversely, the two groups had similar intakes of refined and whole-meal pasta and biscuits as well as breakfast cereals and cakes. Finally, in the control group, a participant consumed whole-meal breakfast cereals and one whole-meal wheat flour, whereas in both groups none consumed other cereals in whole-meal form.

### 2.2. Mycotoxin Excretion

All the 105 urinary samples were screened for the presence of different mycotoxins—such as DON, ZEN, and FB1—as for their main known metabolites (DON-GlcA and DOM-1 for DON; ZEN14GlcA, α and βZEL, and ZEN14Sulf for ZEN). Among all the collected samples, 36 samples (15 control subjects and 21 celiac patients) were positive for the presence of at least one of the considered analytes. The concentration of the analytes was expressed as excretion flow (µg of mycotoxin and/or mycotoxin derivative excreted over 24 h). This calculation was possible by taking into account the total urine volume measured and registered by each participant. Data reporting mean, standard error, median, range, and frequency are summarized in Table 3. In addition to that, also the average excretion values expressed as urinary concentration (µg/L) were reported.

Deoxynivalenol and its metabolite DON-GlcA were found in both groups, with comparable frequency and concentration level. Among the control group, 5 out of 50 urinary samples were found positive to DON (LOD-3.91 µg/day). In four of these samples, the major metabolite DON-GlcA was detected (LOD-8.75 µg/day). DON was also found in four samples belonging to the celiac group (LOD-14.31 µg/day) as its glucuronide form (0.55–10.98 µg/day).

Concerning ZEN and its major phase I metabolites, α- and β-ZEL, urinary concentrations were statistically comparable between groups, although ZEN was found more frequently in urine from celiac patients (14 out of 55) than from control subjects (5 out of 50). 

The other ZEN metabolite, ZEN14GlcA, was detected in only two samples from control subjects. Also FB1 was observed only in the urinary samples collected from the control participants. Other mycotoxin derivatives, as DOM-1 and ZEN14Sulf, were never detected. 

For statistical comparison, data were expressed on a molar base, in terms of DON, ZEN, and FB1 equivalents (as the sum of the parent compound and its metabolites as nmol/day). Data were then compared assuming a non-gaussian distribution, as reported in Table 4. Both lower bound (LB) and upper bound (UB) approaches were applied. In the case of FB1, both the approaches indicated a significant higher urinary concentration in control subjects than in celiac patients. Conversely, using only the UB approach the urinary concentration of DON and ZEN equivalents was significantly higher in celiac patients than in control subjects (Wilcoxon-Mann-Whitney test).

## 3. Discussion

This study reported the level of mycotoxin/mycotoxin metabolite excretion of two groups of subjects, patients affected by celiac disease and non-celiac subjects, both in a free-living diet context. The total number of recruited subjects is limited, but the results obtained are considered as a good starting point to a comparison between celiac patients and control subjects. The study was based on direct monitoring of major urinary biomarkers of exposure to DON, ZEN, and FB1. These analytes were selected on the basis of several considerations. Firstly, this study, in pursuing the previous one, is aimed to evaluate the possible higher exposure of celiac patients to fumonisins, due to the higher corn intake in the diet than the general population [14]. Secondly, since critique related to the EU regulation on mycotoxins in corn-based products points to the lack of differentiation for gluten free products, we have extended the present survey to include also other mycotoxins often found in corn. Therefore, besides FB1, our attention was focused on DON and ZEN. DON has been proven to impair gastrointestinal mucosa [25], while ZEN is a suspected endocrine disruptor worthy of investigation in biomonitoring studies [6].

According to the previous literature, DON, DON-GlcA, and DOM-1 were monitored as biomarkers for dietary DON exposure, ZEN, α- and β-ZEL, ZEN14GlcA, and ZEN14Sulf for dietary ZEN exposure, and FB1 for dietary FB1 exposure. Unfortunately, under the applied chromatographic conditions, DON3Glc and DON15Glc coelute, and therefore the quantification was given as the sum of both peaks. As a consequence, due to the lack of proper quantification of DON15GlcA, the major human metabolite of DON [26], an underestimation of DON exposure cannot be ruled out. 

Based on the results, biomarkers of exposure were found in 15 control subjects and 21 celiac patients, corresponding to about the 34% of the total participants. The co-exposure to DON and ZEN was observed in 3 out of 36 positive samples.

Regarding the daily intake of cereal-based foods, they were very similar between the two groups of participants, with the exception of breads, flours, other cereals, and rice. Intakes were also found to be in line with those measured in the general Italian adult population [1] except for those of sweet products (i.e., biscuits, breakfast cereals, and cakes) that were higher in our volunteers. However, comparing the consumption of such products in both control subjects and celiac patients, this was in agreement with that measured in our previous surveys [14,25,27]. As expected, but in disagreement with our previous study [14], celiac patients had different dietary habits with respect to control subjects in terms of consumption of breads, other cereals, and rice. Compared to control subjects, the higher consumption of rice in the celiac patient’s diet is likely due to the fact that rice is a familiar alternative GF cereal to wheat-based products in the Italian diet, whereas the lower consumption of bread is probably linked to the still low sensory characteristics of GF breads [28]. Overall, the total consumption of cereal-based products is significantly different among the two groups (*p* = 0.021).

According to our data, the urinary excretion of DON and ZEN did not differ among celiac patients and control subjects when an LB approach was used, whereas the two groups showed a significantly different exposure under a UB approach. This is probably due to the possible bias introduced by highly frequent left-censored data in an upper bound approach. 

Considering DON urinary excretion, data presented herein are in line with those reported by Solfrizzo et al. [29], based on a normal-diet cohort from Southern Italy, and with those reported by Heyndrickx et al. for a Belgian population [4]. Our findings are thus consistent with a low but widespread occurrence of DON in cereal-based products. Although slightly higher in the control group, the total cereals intake of the two groups was in an overlapping range. According to the European Union, gluten-containing and GF products should accomplish the same legal limits [30], and regulation does not differentiate between cereals. 

According to Warth et al. [31], a significant difference in DON urinary excretion can be found in people who consume cereal or cereal-based foods in at least one meal per day compared to people following a cereal-free (though not gluten free) diet. Therefore, in consideration of the inter-individual variability in uptake, metabolism, and elimination of mycotoxins, a similar intake likely leads to a comparable exposure, and thus a comparable excretion. 

As far as ZEN is concerned, the parent compound and its metabolites were found in the urine of 5 out of 50 control subjects and in 14 out of 55 celiac patients. The higher frequency is reflected in a significant difference in terms of urinary excretion only when the UB approach is considered, since the high number of left-censored data strongly affects the variability. According to the dietary records and in consideration of the widespread occurrence of ZEN in food commodities and ingredients, it was impossible to link the ZEN exposure to a specific food. In comparison to our results, Heyndrickx et al. [4] did not reporte the occurrence of ZEN biomarkers in urines from its Belgian population, with only one exception. On the contrary, ZEN and its metabolites were reported by Solfrizzo et al. [29]. Besides the differences in analytical sensitivity and sample treatment, this discrepancy could be explained taking into consideration the higher consumption of cereal-based foods and whole-grains in the Mediterranean area compared to Continental Europe [6]. It has been recently reported that ZEN and its modified forms mostly occur in wholegrain and fiber-enriched products [9]. Recommendations to increase consumption of wholegrain and fiber-rich foods are global, due to their well-known health benefits. As a consequence, it could be of special importance to implement dedicated monitoring plans for wholegrain and bran-enriched products, to avoid an increase of exposure to mycotoxins. 

Fumonisins are major contaminants of corn and ingredients thereof, which are frequently used in the GF diet. For this reason, people suffering from gluten-related diseases, obliged to follow a strict gluten-free diet, should be carefully monitored as they could be more exposed to fumonisin contamination than the general population [13,14]. 

In our previous study on a small cohort recruited in the Emilia-Romagna region [14], the estimation of dietary exposure, based on FB1 occurrence data and dietary habits, was higher in celiac patients than in the control group. However, when the sphinganine-to-sphingosine ratio in urine was monitored as indirect biomarker of exposure, no significant difference between celiac patients and control subjects was found. The result was in agreement with the literature when low-exposure population groups are considered, since the basal level of sphingoid suffers from high interindividual variability and, thus, low disruption effects can be lost.

As a possible alternative biomarker, the direct monitoring of urinary FB1 was recently proposed [19,32]. Nonetheless, due to the very low bioavailability of FB1 and its short half-life [33], its detection in the urine of low-exposure subjects is challenging. 

In the present study, FB1 was never detected in the urine from celiac patients, while 3 out of 50 control subjects were found positive. This lead to a significant difference in exposure among groups under both LB and UB models. Taking into consideration that FB1 is poorly bioavailable giving rise thus to a low urinary excretion, the presence of FB1 in three urine samples might be regarded as the possible result of an eventual single dose exposure before urine collection. Unfortunately, it cannot be explained from dietary information obtained in our study. Therefore, this result highlights the importance of extensive monitoring plans for food and food ingredients.

## 4. Conclusions

The present study reports on the exposure to some mycotoxins of a group of celiac patients recruited in Northern Italy, compared to a control group. The exposure was evaluated by monitoring the urinary biomarkers of those mycotoxins often found in corn (i.e., DON, ZEN, and fumonisin B1). Despite the limited number of subjects considered for this study and the absence of actual data on mycotoxin intake due to the lack of mycotoxins content of food, the strengths of the present study are the detailed dietary information collected by a food consumption diary and the analyses carried on a sample of 24 h urines. Using an upper bound but not a lower bound approach, our results indicate that celiac patients have a higher urinary excretion of DON and ZEN. On the other hand, FB1 excretion is more frequent in control subjects (both UB and LB models). However, due to the relevant number of left-censored data leading to a bias in variability evaluation, we found it more appropriate to consider the exposure between the two groups as comparable. 

On one hand, our findings suggest that there is no urgency of specific regulation for gluten free products; on the other hand, a constant surveillance is needed because one-third of the volunteers from both groups recruited during the study tested positive for urinary biomarkers for the considered mycotoxins.

## 5. Experimental Section

### 5.1. Chemicals

Ethyl acetate, acetonitrile, methanol, formic acid, acetic acid, all HPLC grade, and magnesium sulphate were purchased from Sigma-Aldrich (Milan, Italy). Bi-distilled water used for UHPLC analyses as for diluting samples was produced in-house by using a Milli-Q System (Millipore, Bedford, MA, USA). Mycotoxin standard solutions of fumonisin B1, deoxynivalenol, deepoxy-deoxynivalenol, zearalenone, α-zearalenol, and β-zearalenol were obtained from Romer Labs (Tulln, Austria). Deoxynivalenol-3-glucuronide and zearalenone-14-glucuronide were synthesized and purified in our laboratory according to the protocol described by Dall’Erta et al. [34].

### 5.2. Subjects and Study Design

For this study, 105 subjects (55 celiac patients and 50 non-celiac subjects) were enrolled in Lombardy and Emilia Romagna regions (North Italy). All the celiac patients were recruited at the Center for Prevention and Diagnosis of Celiac Disease at the University of Milan as previously described [25], while the non-celiac persons were voluntaries enrolled among students, researchers and professors of the Universities of Parma and Milan. The local Ethical Committee for Human Research of the City of Milan approved the protocol. The study was registered at ClinicalTrials.gov (ID NCT01975155). The exclusion criteria for celiac patients were: diagnosis of CD of less than two years; age younger than 18 years or older than 70 years; diagnosis of metabolic or chronic diseases (e.g., diabetes mellitus, Crohn’s disease, cardiovascular and neurovascular diseases, cancer, neurodegenerative diseases, and rheumatoid arthritis); pregnancy or lactation; and strict vegetarianism. The same exclusion criteria were applied for control subjects, with the exception of the diagnosis of celiac disease. All individuals were recruited between October 2012 and August 2014 and the dietary data were collected during the same period. After having signed a written informed consent, participants were enrolled in the study. During the first visit, they received the instructions on how to record data on food consumption on a seven-day food record diary and a flask for collecting all the urine produced in a 24 h period on the seventh day of the study. 

Total food and beverage consumption was assessed by means of the food consumption diary compiled daily for a total of seven days, as previously described [25]. Participants were asked to weigh all food and drink consumed and to provide a detailed description of each food, including methods of preparation and recipes used, whenever possible. In the case of GF foods, participants were asked to precisely note the name of the manufacturer or to provide the food label. Celiac patients returned their completed seven-day weighed food record diary and the 24-h urine flask during a second visit for at Center for Prevention and Diagnosis of Celiac Disease, whereas control subjects did the same at the Department of Food Science of the University of Parma. The same dietitian reviewed the diaries and, when had concerns regarding possible errors or omissions, contacted by phone the participants to clarify the issues. Dietary data were elaborated using a Microsoft Access application (version 2003, Microsoft Cor. Redmond, WA, USA) linked to the European Institute of Oncology’s food database, covering the nutrient composition of >900 Italian foods [35], integrated with the nutrient composition of 60 GF foods present in the Italian market [36].

The output consisted of the daily intake of energy and food items for each subject. Food items consumed were grouped according to eight food categories: pasta and whole-meal pasta; breads and whole-meal breads (including crackers and salty snacks); breakfast cereals and whole-meal breakfast cereals; biscuits and whole-meal biscuits; cakes; rice and brown rice; other cereals and other whole-meal cereals (including wheat, corn, oat, quinoa, and buckwheat) all consumed as grains; cereal flours and whole-meal flours (including wheat, corn, oat, quinoa, rice, and buckwheat). 

### 5.3. Urine Sample Collection and Extraction

The 24-h urine was collected for each participant during the last day of the dietary monitored week. All participants were instructed to record the total volume of urine using the scale on the graduated flask. Samples were divided in 40 mL aliquots and stored at −80 °C until analysis.

Mycotoxin extraction was performed on two aliquots of each sample, applying the protocol described by Song et al. in 2013 [37]. Briefly, 5 mL of urine were mixed with 10 mL of a magnesium sulphate aqueous solution (2M) and extracted with 5 mL of ethyl acetate added with formic acid (1%) on a shaker (200 stokes/min, room temperature, 15 min). After that, the sample was centrifuged for 15 min at 3220 rcf at 25 °C and the resulting supernatant was removed and kept in a Falcon tube. An additional extraction step was performed on the sample adding 5 mL of acetonitrile acidified with formic acid (1%) following the same procedure adopted for the first extraction. Then, the two organic phases were pooled and dried under a slight nitrogen flow at room temperature. The dried extract was dissolved with 0.5 mL of acidified bi-distilled water (0.2% of formic acid) and then analyzed by UPLC-MS/MS.

### 5.4. UHPC-ESI-MS/MS Analysis

Urine extracts were analyzed on a UHPLC (Dionex UltiMate 3000) apparatus coupled with a triple quadrupole (TSQ Vantage) (Thermo Fisher Scientific Inc., San Jose, CA, USA) equipped with an ESI interface. For analyte separation a RP-C18 Kinetex column (2.6 µm, 100A; 100 × 2.10 mm; Phenomenex, Torrance, CA, USA) was used. Mobile phases were composed of aqueous ammonium acetate (0.5 mM) (A) and methanol (B) both acidified with 0.2% of acetic acid. A gradient elution system was applied: from 2% of B at time zero to 20% of B in 0.2 min, these conditions were maintained for 7.8 min, then % of B increased to 90% in 7 min, the column was flashed under these conditions for 5 min and then initial proportions were re-established with a resulting total run time of 30 min. The flow was maintained at 0.35 mL/min during the run and 2 µL of each sample were injected. 

All the mycotoxins, except fumonisin B1, were detected in negative ionization mode applying a spay voltage of 3500 V, a capillary temperature of 270 °C, a vaporizer temperature of 200 °C and a sheath gas flow of 50 units. For fumonisin B1, the same parameters were applied but in positive mode. The other parameters for mycotoxin determination, such as S-Lens RF amplitude values, were obtained and set by tuning methanolic solutions of each analyte considered (1 mg/kg). Multiple reaction monitoring (MRM) modality was adopted for mycotoxin detection and the following transitions were monitored: 355.2 → 265 (CE = 17 eV), 355.2 → 295 (CE = 13 eV) for DON; 471.2 → 175.2 (CE = 23 eV), 471.2 → 265.2 (CE = 13 eV) for DON-3-GlcA; 339.2 → 59 (CE = 35 eV), 339.2 → 249 (CE = 17 eV) for DOM-1; 317.2 → 131 (CE = 34 eV), 317.2 → 175 (CE = 28 eV) for ZEN; 319.1 → 129.9 (CE = 48 eV) for αZEL and βZEL; 397.2 → 131 (CE = 24 eV), 397.2 → 175 (CE = 24 eV), 397.2 → 317.1 (CE = 24 eV) for ZEN-14-S; 493.2 → 131 (CE = 45 eV), 493.2 → 175 (CE = 21 eV), 493.2 → 317.1 (CE = 28 eV) for ZEN-14-GlcA; 722.4 → 334.1 (CE = 39 eV), and 722.4 → 352.2 (CE = 35 eV) for FB1.

**Figure 1:**
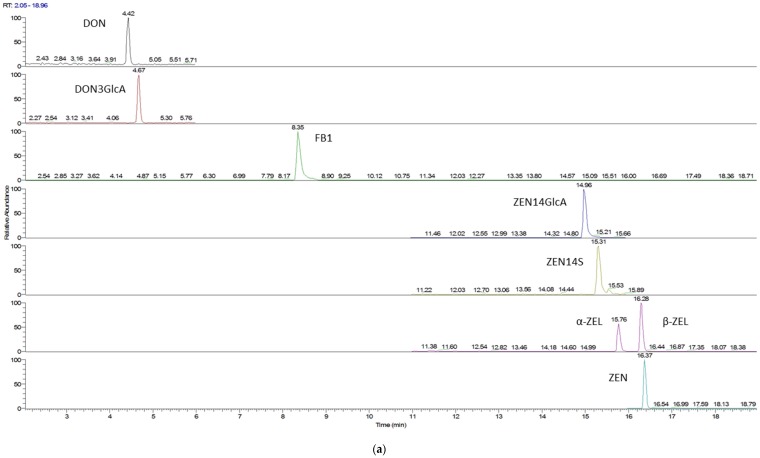

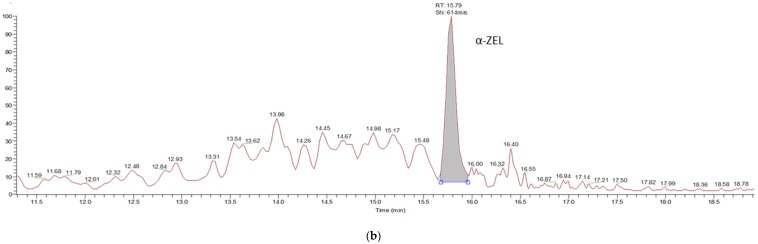
Chromatographic separation of a blank sample spiked with standard mixture (**a**); and of urine sample contaminated by α-ZEL (**b**).

For each analyte considered, selectivity limit of detection (LOD) and limit of quantification (LOQ) were obtained using not-contaminated urine as blank. Selectivity was assessed by checking the absence of interferences for the chosen mass transitions. Moreover, the detection of the different mycotoxins was performed dividing the run in three different time segments on the basis of the analyte retention times: in the first segment the transitions of DON, DOM-1, and DON-3-GlcA were monitored, in the second those of FB1, while in the third those of ZEN and its derivatives. Values for LOD and LOQ were 0.32 and 1.07 µg/L for DON, 0.13 and 0.43 µg/L for DON-GlcA, 0.003 and 0.009 µg/L for ZEN, 0.09 and 0.30 µg/L for ZEN14GlcA, 0.03 and 0.09 µg/L for ZELs, 0.05 and 0.17 µg/L for FB1. A stock solution at the concentration of 50 µg/L of all considered analytes was prepared using a blank urine extract as solvent. Then, calibration curves were obtained by diluting the stock solutions at five different concentration levels in the range 10–200 µg/L. Recovery rate percentages (RR%) were calculated for DON, ZEN, αZEL, and βZEL and FB1 by extracting and analyzing one blank urine sample spiked with a concentration of 50 µg/L of each analyte. All the RR% values were in the range 90%–100%. Recovery was slightly lower for conjugates (DON-Glc, ZEN14Glc) in consideration of the higher polarity of these compounds (RR% > 80%). A chromatogram of standard mixture and of a urine sample containing α-ZEL is reported in Figure 1.

### 5.5. Statistical Analysis

Data were analyzed following both the lower bound and the upper bound approach, using LOD in the latter for left-censored samples. In consideration of the non-gaussian distribution of the data, a Wilcoxon-Mann-Whitney non parametric test was used for group comparison. Analysis was carried out using IBM SPSS Statistics 23.0.0 (IBM, Milan, Italy). 

## Figures and Tables

**Table 1:** Baseline characteristics and daily energy intake of celiac patients and control subjects.

Characteristics	Celiac Patients (*n* = 55)	Control Subjects (*n* = 50)
Age ^a^, year	43.2 ± 13.2	38.4 ± 13.6
Body Mass Index ^a^, kg/m^2^	22.8 ± 4.1	22.5 ± 3.9
Energy Intake ^a^, kcal/day	2130 ± 281	2132 ± 309
Female, *n* (%)	42 (76)	37 (74)

^a^ Values are mean ± SD.

**Table 2:** Mean ± SE, median, range (min–max), expressed as g/day, of daily intake of analyzed food items for celiac patients and control subjects.

Food Categories (g/day)	Celiac Patients (*n* = 55)			Control Subjects (*n* = 50)			*p*-Value ^a^
	Mean ± SE	Median	Range	Mean ± SE	Median	Range	
Bread and substitutes	84.5 ± 7.0	76.0	1.4–215.7	125.9 ± 7.6	112.8	15.0–268.6	0.000
Whole-meal bread and substitutes	3.3 ± 1.7	0.0	0.0–72.9	10.6 ± 2.7	0.0	0.0–74.3	0.000
Flour	3.7 ± 1.0	0.0	0.0–31.4	8.8 ± 1.7	3.6	0.0–48.0	*0.003*
Pasta	47.5 ± 4.4	44.3	0.0–167.1	51.1 ± 4.3	47.9	0.0–131.4	0.460
Whole-meal pasta	0.8 ± 0.5	0.0	0.0–22.9	2.20 ± 1.1	0.0	0.0–41.4	0.361
Rice	24.2 ± 2.8	20.9	0.0–81.4	13.7 ± 2.0	10.0	0.0–52.9	*0.010*
Brown rice	1.0 ± 0.6	0.0	0.0–22.9	1.11 ± 0.5	0.0	0.0–17.1	0.419
Breakfast cereals	9.5 ± 2.9	0.0	0.0–98.6	5.3 ± 1.9	0.0	0.0–83.6	0.402
Biscuits	23.7 ± 3.2	18.1	0.0–105.0	21.2 ± 3.1	12.0	0.0–107.1	0.744
Whole-meal biscuits	0.8 ± 0.5	0.0	0.0–20.0	2.0 ± 0.8	0.0	0.0–32.9	0.060
Cakes	32.1 ± 5.2	17.1	0.0–180.0	34.0 ± 4.0	26.5	0.0–114.3	0.259
Other cereals	19.8 ± 3.7	5.7	0.0–112.9	11.0 ± 2.7	0.0	0.0–75.7	*0.043*
Total cereal-based food	252.9 ± 9.1	248.3	123.3–411.3	286.1 ± 9.3	273.9	189.8–442.3	*0.021*

^a^ Comparisons were performed using the Wilcoxon-Mann-Whitney non-parametric test.

**Table 3:** Mean ± SE, median, range (min–max), both expressed as µg/day, and frequency (% of positive subjects on the total: 50 for control subjects and 55 for celiac patients), of urinary biomarkers detected in the study participants. Mean and median values were calculated considering all the samples; left-censored values were considered using a lower bound approach.

Groups		DON	DON-GlcA	ZEN	α+βZEL	ZEN14GlcA	FB1
**Control subjects**	Mean ± SE (µg/day)	0.92 ± 0.42	1.37 ± 0.79	0.01 ± 0.01	0.06 ± 0.04	0.06 ± 0.06	0.19 ± 0.12
	Mean ± SE (µg/L)	0.17 ± 0.08	0.23 ± 0.13	0.03 ± 0.00	0.09 ± 0.01	0.02 ± 0.01	0.07 ± 0.05
	Median (µg/day)	0.00	0.00	0.00	0.00	0.00	0.00
	Median (µg/L)	0.00	0.00	0.000	0.002	0.000	0.00
	Range (min–max) (µg/day)	LOD *–3.91	LOD *–8.75	LOD *–0.08	LOD *–0.50	LOD *–0.83	LOD *–1.52
	Range (min–max) (µg/L)	LOD *–2.41	LOD *–5.84	LOD *–0.05	LOD *–0.25	LOD *–0.83	LOD *–2.54
	Freq. (%) (positive/total)	10 (5/50)	12 (6/50)	10 (5/50)	4 (2/50)	4 (2/50)	5 (3/50)
**Celiac patients**	Mean ± SE (µg/day)	1.09 ± 0.76	1.42 ± 0.72	0.04 ± 0.01	0.02 ± 0.12	0.00	0.00
	Mean ± SE (µg/L)	0.22 ± 0.17	0.22 ± 0.13	0.01 ± 0.00	0.06 ± 0.04	0.02 ± 0.01	0.07 ± 0.05
	Median (µg/day)	0.00	0.00	0.00	0.00	0.00	0.00
	Median (µg/L)	0.00	0.00	0.002	0.002	0.000	0.00
	Range (min–max) (µg/day)	LOD *–14.31	LOD *–10.98	LOD *–0.22	LOD *–2.19	-	-
	Range (min–max) (µg/L)	LOD *–8.94	LOD *–5.59	LOD *–0.10	LOD *–2.19	-	-
	Freq. (%) (positive/total)	7 (4/55)	9 (5/55)	25 (14/55)	9 (5/55)	0 (0/55)	0 (0/55)

* LODs: 0.32 µg/mL for DON; 0.13 µg/mL for DON-GlcA; 0.003 µg/mL for ZEN; 0.09 µg/mL for ZEN14GlcA; 0.03 µg/mL for ZELs; 0.05 µg/mL for FB1.

**Table 4:** Mean ± SE, median, range (min–max), expressed as µmol equivalents/day for DON an ZEN and in nmol equivalents/day for FB1, of urinary biomarkers detected in the study participants. Mean and median values were calculated considering all the samples; both lower and upper bound approaches were used for left-censored data.

Group		Lower Bound			Upper Bound		
		DON (µmol eq/day)	ZEN (µmol eq/day)	FB1 (nmol eq/day)	DON (µmol eq/day)	ZEN (µmol eq/day)	FB1 (nmol eq/day)
**Control subjects**	Mean ± SE	0.22 ± 0.14	0.03 ± 0.02	0.07 ± 0.05	0.39 ± 0.17	0.09 ± 0.01	0.14 ± 0.04
	Median	0.00	0.00	0.00	0.12	0.07	0.07
	Range (min–max)	0.00–5.50	0.00–0.54	0.00–2.11	0.11–7.21	0.07–0.59	0.07–2.11
**Celiac patients**	Mean ± SE	0.26 ± 0.16	0.07 ± 0.04	0.00 ± 0.00	0.44 ± 0.21	0.14 ± 0.04	0.07 ± 0.00
	Median	0.00	0.00	0.00	0.11	0.07	0.07
	Range (min–max)	0.00–6.89	0.00–2.17	-	0.11–9.03	0.07–2.24	0.07–0.07
***p*-value ^a^**		n.s.	n.s.	0.033	0.000	0.000	0.033

^a^ Comparisons were performed using the Wilcoxon-Mann-Whitney non-parametric test; n.s., not significant (*p* > 0.05).
